# Tuina combined with Riluzole in amyotrophic lateral sclerosis: protocol for a randomized controlled trial with clinical outcomes and synaptic PET biomarkers

**DOI:** 10.3389/fneur.2025.1705466

**Published:** 2025-11-10

**Authors:** Dan Yang, Jing Zhou, Yan Zhao, Mohammad Nasb

**Affiliations:** 1 Rehabilitation Medicine Center/Tuina Department, Hubei Provincial Hospital of Traditional Chinese Medicine, Wuhan, China; 2 Affiliated Hospital of Hubei University of Chinese Medicine, Wuhan, China; 3 The First Clinical Medical School, Hubei University of Chinese Medicine, Wuhan, China; 4 Hubei Shizhen Laboratory, Wuhan, China; 5 Hubei Key Laboratory of Theory and Application Research of Liver and Kidney in Traditional Chinese Medicine (Hubei Province Hospital of Traditional Chinese Medicine), Wuhan, China; 6 Health Science Faculty, Homs University, Homs, Syria

**Keywords:** amyotrophic lateral sclerosis, Chinese medicine, Tuina therapy, comprehensive rehabilitation, quality of life

## Abstract

**Background:**

Amyotrophic lateral sclerosis (ALS) is a devastating neurodegenerative disease characterized by progressive motor neuron degeneration, leading to severe functional decline and limited therapeutic options. While current pharmacological interventions such as Riluzole offer only modest benefits, there is a growing imperative to explore complementary rehabilitation strategies. Preclinical and neuroimaging evidence suggests that Tuina, a traditional Chinese manual therapy, may influence synaptic plasticity and integrity, offering a biologically reasonable mechanism for therapeutic benefit.

**Methods:**

This randomized controlled trial, approved by the Ethics Committee of Hubei Provincial Hospital of Traditional Chinese Medicine (Approval No. HBZY2022-C42-01), will use a 1:1:1 allocation to enroll 135 participants. Participants will be assigned to: (i) Tuina therapy plus oral Riluzole, (ii) sham Tuina plus oral Riluzole, or (iii) Riluzole alone. Interventions will last for 1 year. The primary outcome is the change in Amyotrophic Lateral Sclerosis Functional Rating Scale-Revised (ALSFRS-R) scores. Secondary outcomes include manual muscle test (MMT), modified Ashworth scale (MAS), forced vital capacity (FVC), vital capacity (VC), forced expiratory volume in 1 second (FEV1), FEV1/FVC ratio, peak expiratory flow (PEF), maximal voluntary ventilation (MVV), and ALS Assessment Questionnaire (ALSAQ-40). Outcomes will be assessed at baseline, 4 weeks, and every 6 months up to 24 months. A mechanistic substudy will employ presynaptic Synaptic vesicle protein 2A (SV2A) PET imaging to quantify synaptic changes associated with Tuina intervention.

**Discussion:**

This study is designed to evaluate the clinical efficacy of Tuina therapy combined with Riluzole and to investigate its potential to modulate synaptic integrity in patients with ALS. The findings are expected to provide evidence for integrating Tuina as an adjunctive, non-pharmacological therapy into comprehensive ALS management, linking functional improvements to underlying synaptic mechanisms.

**Clinical trial registration:**

https://www.chictr.org.cn, identifier ChiCTR2300068650

## 1 Introduction

ALS is a persistently progressive and fatal neurodegenerative disease marked by the degeneration of both upper and lower motor neurons in the motor cortex, brainstem, and spinal cord (1). This neuronal loss results in characteristic symptoms such as progressive muscle weakness, atrophy, fasciculations, and spasticity, ultimately leading to profound impairment of voluntary muscle control (2). A comprehensive systematic review identified 6,238 articles, of which 140 met the inclusion criteria for data extraction. Among these, 85 studies reported ALS incidence and 61 reported prevalence. The incidence rates varied widely, ranging from 0.26 to 23.46 per 100,000 person-years, with the lowest observed in Ecuador and the highest in Japan. Point prevalence ranged from 1.57 to 11.80 per 100,000 individuals. Notably, many of the included studies employed multiple data sources to enhance case ascertainment and diagnostic accuracy (3).

The rapid and progressive decline in motor function in ALS patients profoundly impairs their capacity to perform basic activities of daily living, including washing, dressing, and ambulation, ultimately leading to a marked loss of independence and a significant deterioration in quality of life (QoL) (4). As the disease advances, involvement of the respiratory muscles becomes inevitable, with respiratory failure representing the primary cause of mortality in affected individuals (5). Additionally, swallowing impairment (dysphagia) is a hallmark feature of ALS. Dysphagia develops in more than 85% of patients and contributes to malnutrition, dehydration, aspiration and poor quality of life (6, 7).

Despite decades of investigation, the etiology of ALS remains largely elusive, and no curative treatment has been identified (8). Current pharmacological options, such as Riluzole, and Edaravone offer only modest benefits, primarily in slowing disease progression and extending survival by a few months (9, 10). Diagnostic delays in ALS are substantial, with a median lag of 10 to 16 months from symptom onset to confirmed diagnosis—equating to nearly one-third of a patient’s remaining lifespan (11). This delay exposes individuals to accelerated motor decline, reducing their ability to perform essential daily tasks such as dressing, bathing, and ambulating, and severely compromising both their quality of life and autonomy (12).

In light of the limited efficacy of current pharmacologic agents, there is growing interest in adjunctive non-pharmacological strategies to support ALS patients. Among these, Tuina, a form of Chinese therapeutic manual therapy, may offer unique advantages. In a case report conducted by the department, a patient with ALS experienced improvements in pulmonary function and quality of life following combined Tuina and Western medicine treatment, suggesting clinical compelling (13). Previous studies suggest that Tuina can promote behavioral improvement and brain plasticity, particularly in cases of peripheral nerve injury (14). It has demonstrated analgesic effects by acting on different levels of neural targets, making it an effective therapy for neuropathic pain (15). Furthermore, Tuina has been shown to influence resting state brain activity, suggesting its ability to modulate asynchronous activity in abnormal brain regions (16). Recent findings also indicate that Tuina therapy can inhibit motor neuron apoptosis and alleviate inflammation in sciatic nerve injury models (17). Its application has been explored in conditions such as Parkinson’s disease, where it shows promise in addressing non-motor symptoms (18), and in improving cognitive functions in hypoxic-ischemic brain injury (19). The mechanisms underlying these neurological benefits may involve nerve myelin regeneration, acceleration of axonal regeneration (20), and modulation of neuroinflammation and microglial activation (21).

A recent network meta-analysis of 96 randomized controlled trials on non-pharmacological interventions for post stroke dysphagia found that combinations including Tuina improved swallowing safety more effectively than single interventions. The analysis noted that Tuina treats post stroke dysphagia by promoting local muscle function recovery and improving the function of the face, throat and larynx (22). Additionally, a case report of a patient with ALS receiving combined Tuina and Western medical treatment observed improvements in pulmonary function and quality of life. After 30 days of daily Tuina therapy, the patient reported improvements in slurred speech, choking on drinking water and tongue motion (13).

These findings underscore Tuina’s potential as a valuable therapeutic modality in the context of neurodegenerative diseases and neurological recovery. By stimulating peripheral and segmental neural circuits, Tuina may exert neuroregulatory effects that support motor neuron integrity and mitigate functional decline. Tuina may support synaptic integrity and circuit-level plasticity in motor networks, precisely the domains affected in ALS. By integrating mechanistic neuroimaging modalities, especially SV2A PET, this trial is uniquely positioned to determine whether Tuina can promote presynaptic structural changes alongside clinical improvements.

## 2 Objectives and hypothesis

Given the rapidly progressive nature of ALS and the limited benefits of current pharmacological interventions, there is an urgent need for adjunctive strategies that may preserve function and improve patient quality of life. Tuina has been proposed as a supportive intervention that may influence neuromuscular performance and central neural function.

The primary objective of this trial is to evaluate whether Tuina therapy combined with Riluzole can slow the decline of physical function in patients with early-stage ALS, as assessed by changes in the ALSFRS-R. Secondary objectives include determining the impact of the combined intervention on respiratory capacity, muscle strength, spasticity, and quality of life, as well as assessing the overall safety and tolerability of repeated Tuina sessions. Beyond clinical outcomes, the trial will also explore potential neurobiological mechanisms of Tuina. Using presynaptic SV2A PET imaging at baseline and 6 months, the objective is to examine whether functional changes are accompanied by preservation or enhancement of synaptic integrity in motor system regions.

It is hypothesize that Tuina combined with Riluzole will significantly slow functional decline and improve quality of life compared with Riluzole alone or sham Tuina plus Riluzole. Furthermore, it is anticipated that these clinical benefits may be linked to measurable synaptic changes, providing mechanistic evidence for Tuina as a complementary therapy in ALS.

## 3 Methods and analysis

### 3.1 Study design

This study is designed as a prospective, parallel-group, randomized controlled trial with a three-arm comparative design. Patients with ALS are identified and enrolled through systematic screening at the Hubei Provincial Hospital of Traditional Chinese Medicine. Recruitment began in September 2024 and is ongoing. Eligible participants will be randomly assigned in a 1:1:1 ratio to one of three groups: (i) Tuina therapy combined with Riluzole, (ii) sham Tuina therapy combined with Riluzole, or (iii) Riluzole alone. Randomization is performed using a computer-generated sequence created by an independent statistician not involved in participant recruitment or outcome assessment. Allocation is concealed using sequentially numbered, opaque, sealed envelopes (SNOSE) prepared prior to enrollment, and the envelopes are opened only after completion of baseline assessments to minimize selection bias.

**Figure 1:**
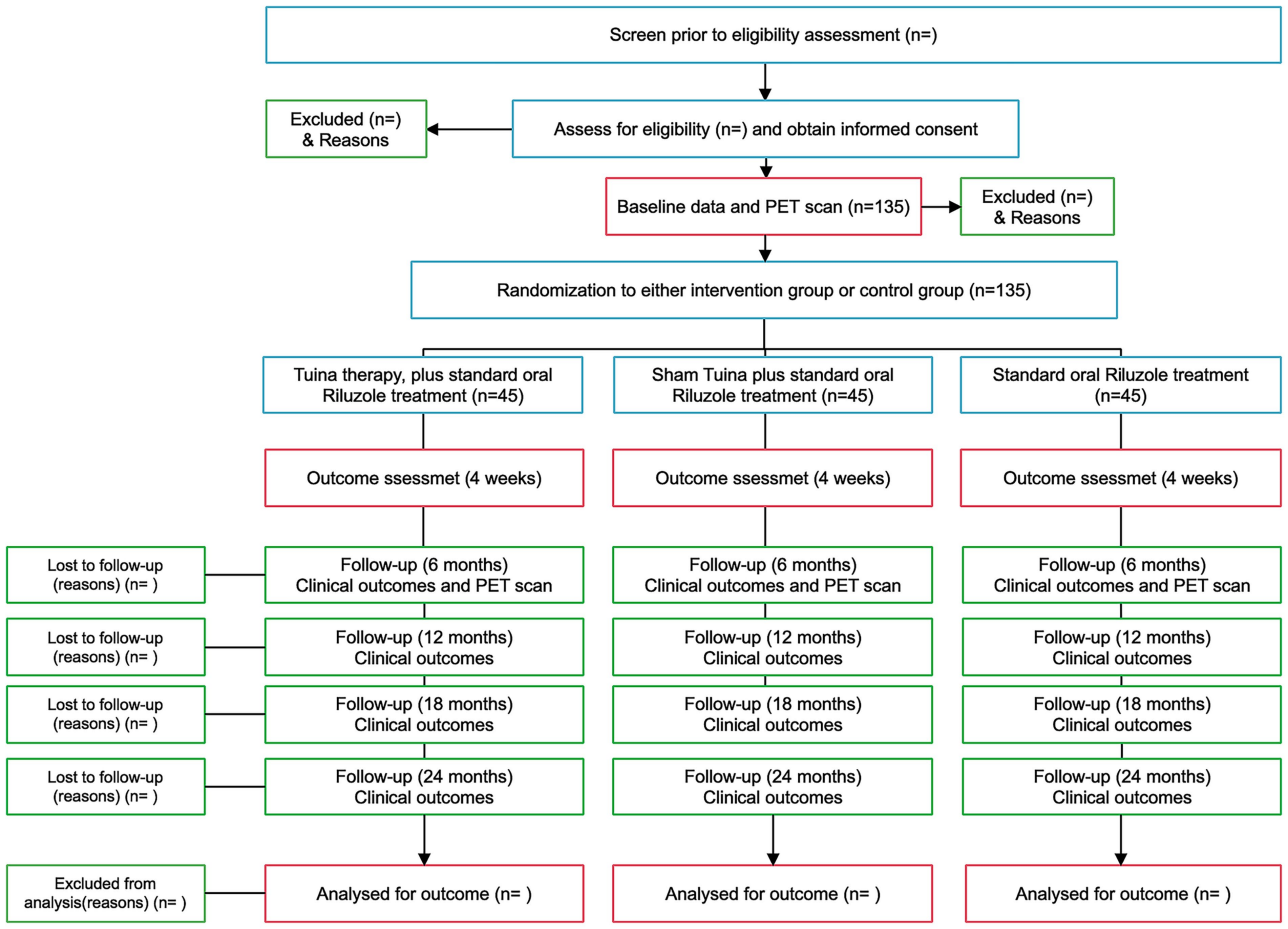
CONSORT flowchart illustrating the stages of the study, including screening, baseline data collection, randomization, outcome assessment, follow-ups, and analysis.

All participants continue to receive standard pharmacological treatment with oral Riluzole throughout the trial. The intervention group receives a structured program of Tuina therapy, while the control group receives sham Tuina matched in duration and frequency. The standard care group receives Riluzole only. Interventions are delivered over a total treatment period of 12 months, beginning with a 4-week intensive phase followed by a maintenance schedule. Clinical efficacy and safety outcomes are assessed at baseline, after the 4-week intensive phase, and at Months 6, 12, 18, and 24. A mechanistic substudy employs presynaptic SV2A PET imaging at baseline and Month 6 to explore synaptic integrity changes associated with Tuina. A schematic flowchart is presented in Figure 1, and the study timeline is summarized in Table 1.

**Table 1:** Study design timeline illustrating enrollment, allocation, interventions, and outcome measurements at specified time points.

Time point	Enrollment	Allocation	Outcome measurement				
		Week 0	4 Weeks	6 Months	12 Months	18 Months	24 Months
Enrollment							
Eligibility screen	●						
Informed consent	●						
Allocation		●					
Interventions							
Riluzole		●	●	●	●	●	●
Tuina therapy		○	○	○	○	◎	◎
Assessments							
Baseline characteristics		●					
ALSFRS-R		●	●	●	●	●	●
Pulmonary function test		●	●	●	●	●	●
MMT		●	●	●	●	●	●
MAS		●	●	●	●	●	●
ALSAQ-40		●	●	●	●	●	●
Adverse event monitoring		●	●	●	●	●	●
PET		●	◎	●	◎	◎	◎

● Common in both groups. ○ Experimental group only. ◎ Neither in two groups.

### 3.2 Sample size

The sample size calculation was primarily based on the ALSFRS-R as the main clinical endpoint, reflecting the study’s primary objective to evaluate functional decline. Calculations were performed using G*Power software (version 3.1.9.7) for an F-test. Based on a projected medium effect size (*f* = 0.35) for the ALSFRS-R, derived from prior research and clinical expectations for interventions in ALS, a statistical power of 90% (1-*β* = 0.9) and a significance level of *α* = 0.05 across three groups, a total sample size of 108 participants was determined to be necessary. This calculation yields an actual power of approximately 0.905, ensuring sufficient power to detect a clinically meaningful effect on the primary outcome.

To account for the inherent heterogeneity of ALS and to enhance the ability to detect moderate effect sizes and explore potential subgroup differences, the final recruitment target has been further adjusted. Considering an anticipated dropout rate of approximately 20% over the 12-month treatment and follow-up period, the required effective sample size of 36 participants per group (108 participants total) necessitates recruiting 45 participants per group. Thus, the total recruitment target for the study is 135 participants (45 participants × 3 groups). This increased recruitment target ensures that, even with expected attrition, the study will retain sufficient power for the primary outcome and will also allow for more reliable detection of moderate effect sizes, thereby enhancing the precision of exploratory subgroup analyses in this complex disease.

### 3.3 Eligibility criteria

To ensure a homogeneous sample regarding initial disease severity and to minimize the impact of disease heterogeneity, stringent eligibility criteria will be applied, drawing inspiration from successful ALS clinical trials. This approach aims to create a cohort with comparable disease progression rates and functional status at baseline, thereby enhancing the internal validity and interpretability of our findings. Participants will be eligible for enrollment if they meet all the following criteria: Participants must have a confirmed diagnosis of definite or laboratory-supported probable ALS according to the revised El Escorial criteria, including both sporadic and familial cases.

Eligible individuals will be between 18 and 80 years of age, regardless of sex, and will be enrolled only if they are within 24 months of symptom onset, a criterion intended to reduce variability in disease progression rates. Participants must present with mild to moderate disability corresponding to Sinaki–Mulder stages I–III. Additionally, at baseline, they will need to have a total ALSFRS-R score between 30 and 40, with a minimum score of ≥2 on each of the 12 individual items; this combined threshold is designed to capture patients with early to moderate disability, avoiding floor or ceiling effects and ensuring the scale’s sensitivity to change. Participants must have a forced vital capacity (FVC) of at least 50% of the predicted value based on age, sex, and height, and specifically an FVC of ≥80% of predicted values. Eligible individuals should demonstrate residual limb muscle strength of grade 3 or higher on the Medical Research Council (MRC) scale. Furthermore, patients will be classified using King’s ALS clinical stages 1–2 to confirm early functional stages based on clinical evaluation.

Finally, all participants must be capable of comprehending and following basic verbal motor instructions and must provide written informed consent prior to enrollment. These comprehensive criteria, encompassing diagnostic confirmation, disease duration, functional assessment (ALSFRS-R and Sinaki-Mulder), respiratory capacity (FVC), muscle strength, and clinical staging, will be applied at screening and confirmed through two assessments spaced 2 weeks apart. This multi-dimensional approach to baseline assessment will establish a patient cohort with controlled initial disease severity, thereby increasing the likelihood that any observed therapeutic effects reflect true intervention efficacy.

While the initial site of symptom onset (e.g., bulbar, limb,) will not be an explicit inclusion/exclusion criterion for sample homogeneity, it is recognized that the onset lesion place can significantly influence disease progression and phenotypic presentation in ALS. Therefore, this variable will be considered as a potential covariate in statistical analyses to adjust for its influence on treatment outcomes. Furthermore, *post-hoc* subgroup analyses will be conducted, if statistically powered, to explore differential treatment effects across various onset types. This approach will allow for a more nuanced understanding of the intervention’s efficacy across different ALS phenotypes without overly restricting participant recruitment at the outset of the study.

Exclusion criteria include the presence of a gastrostomy tube or the need for invasive ventilation, a history of other neurodegenerative diseases, or neurological conditions that mimic ALS or could interfere with outcome assessments, such as dementia or cervical spondylotic myelopathy. Individuals with significant comorbidities, including cardiac, pulmonary, hematological, endocrine, or psychiatric disorders, will be excluded if these conditions are judged likely to confound trial outcomes or compromise safety. Women who are pregnant or breastfeeding will not be eligible. Additional exclusions include prior participation in another interventional trial within 3 months before screening, and severe or unstable medical conditions that, in the judgment of investigators, would render the patient unsuitable for Tuina therapy or Riluzole treatment.

### 3.4 Interventions

All participants, regardless of group allocation, will continue standard pharmacological treatment with oral Riluzole at a dose of 50 mg twice daily throughout the study period. Participants in the intervention arm will additionally undergo a structured program of Tuina therapy. This program will consist of sessions delivered over 12 consecutive months (five sessions per week). Each session will last approximately 45 min and will be administered by a certified senior Tuina therapist.

A tailored approach to Tuina therapy is essential for addressing the specific neuromuscular impairments associated with different segments of the body. According to the neuromuscular segmental localization principle in both traditional Chinese medicine and modern neuroanatomy, Tuina therapy can be strategically applied to areas where damage to motor neurons or muscular function occurs. By focusing on specific segments of the body, Tuina can provide precise stimulation that targets the dysfunction of corresponding spinal segments or neuroanatomical regions, optimizing therapeutic outcomes.

In Chinese medicine, this concept of segmental damage aligns with the idea that Qi and blood flow in specific meridians or channels are disrupted as a result of nerve dysfunction. The motor innervation corresponding to each spinal segment directly influences the muscles and joints within its dermatome or myotome. Thus, Tuina can be applied in a targeted manner to specific body segments corresponding to the site of neuronal damage, enhancing the treatment’s efficacy. Tuina therapy should be performed with a focus on the specific body segment affected by ALS, based on the neurological localization of motor neuron degeneration. The following table outlines common neuromuscular segments typically involved in ALS and the corresponding Tuina techniques (Table 2).

**Table 2:** Tuina therapy techniques for addressing neuromuscular impairments in ALS patients.

Step	Technique	Area of focus	Purpose	Description
1	Head and neck massage	Head, neck, face	Relieve tension	Gentle pressure on key acupoints like Yintang (between the eyebrows), Taiyang (temple), and Fengchi (back of the skull). This promotes blood circulation to relieve headache, tension, and improve neurological function.
2	Shoulder and arm manipulation	Shoulders, upper arms	Reduce spasticity	Kneading, pressing, and rolling to alleviate tight muscles and improve motor function in the upper limbs, crucial for ALS-related muscle wasting.
3	Back and spine technique	Spine, shoulder blades, ribs	Enhance flexibility and circulation	Rubbing and kneading along the back and spine stimulate muscle relaxation and improve respiratory function. Focus on Du Mai (Governing Vessel) acupoints for energy flow and pain relief.
4	Lower limb treatment	Legs, feet, and joints	Improve mobility and circulation	Gentle pressing, kneading, and shaking to stimulate muscle function in the lower extremities, aiding in motor coordination and muscle recovery. Target key acupoints for Qi circulation like Zu San Li (Stomach 36) and Shen Shu (Kidney 23).
5	Abdominal and chest massage	Abdomen and chest	Regulate internal organs, improve breathing	Circular motions on the abdomen to boost digestion and percussion techniques on the chest for respiratory strengthening. This is especially important in ALS patients with respiratory failure.
6	Advanced joint mobilization	Ankles, knees, elbows	Restore function, prevent contractures	Gentle joint manipulation through rotation and stretching to prevent joint stiffness and increase range of motion.

Tuina will be adapted not only to the spinal segment affected but also to the stage of ALS. In the early stages, when ALS predominantly affects upper motor neurons (spasticity, muscle weakness), Tuina techniques should focus on gentle manipulation to alleviate muscle tension and improve joint flexibility. In the advanced stages, when lower motor neuron involvement leads to muscle atrophy and flaccidity, Tuina should emphasize deep tissue stimulation to enhance blood circulation and prevent further atrophy.

The personalization of Tuina based on neuromuscular localization allows for localized stimulation of affected motor neurons through targeted manual pressure, which improves the transmission of neural signals. It also enhances muscle flexibility by mobilizing joints and soft tissues around the affected segment, and activates synaptic plasticity in motor pathways through consistent, targeted application of Tuina, ultimately promoting neuromuscular reorganization.

Tuina therapy should be continuously adapted in response to the patient’s evolving symptoms and neurological feedback. For instance, increased spasticity in the early stages requires techniques focused on muscle relaxation and softening, while muscle wasting in the later stages necessitates techniques aimed at increasing muscle tone and enhancing muscle strength. Regular assessments of the patient’s muscle strength, muscle tone, and joint mobility should guide modifications to the manual techniques used, ensuring they align with the patient’s specific neuromuscular needs. By incorporating neuromuscular segmental localization into Tuina therapy, we can offer a targeted, individualized treatment approach that maximizes therapeutic benefits for ALS patients.

During the consent process, participants will receive a clear explanation of the intervention protocol, including session structure, treatment goals, and the importance of consistent attendance. This information will be reinforced through a participant handbook outlining the treatment schedule, simplified descriptions of Tuina therapy, and contact details for study staff to support adherence and address participant concerns.

Participants assigned to the sham Tuina group will receive a structured program designed to mimic the duration, frequency, and therapist–patient interaction of active Tuina therapy, without applying therapeutic pressure or stimulation of acupoints administered by a certified Tuina therapist trained to deliver both active and sham protocols. The sham intervention will include light, non-penetrating contact such as gentle gliding and static touch performed along non-meridian areas, deliberately avoiding recognized acupoints and therapeutic manipulations. Pressure will be minimal and insufficient to elicit physiological or neuromuscular responses.

To ensure credibility, participants will be informed that they are receiving a form of manual therapy under investigation for its potential effects in ALS, without specifying whether their allocation represents active or sham treatment. Treatment fidelity will be maintained through session checklists, random supervisory observations, and regular therapist training. At selected time points, participants will complete a treatment credibility and expectancy questionnaire to confirm that sham Tuina is perceived as an acceptable intervention.

### 3.5 Follow-up

After completion of the 4-week treatment phase, all participants will undergo scheduled follow-up assessments at 6-month intervals over a total duration of 2 years, resulting in four follow-up visits per patient. Follow-up evaluations will include all predefined outcome measures to monitor long-term effects and disease progression, including the ALSFRS-R, manual muscle test (MMT), forced vital capacity (FVC), and other secondary outcomes. Participants who cannot be reached after five consecutive days of contact attempts by the follow-up nurse will be classified as lost to follow-up.

### 3.6 Outcome measurement

Each participant will undergo assessments at baseline (T0), immediately after the 4-week intervention, and at follow-up visits scheduled at 6, 12, 18, and 24 months. These repeated measures are designed to capture both short-term effects of the intervention and its longer-term influence on disease progression, functional capacity, and quality of life. The primary outcome is the change in global functional status, measured using the Amyotrophic Lateral Sclerosis Functional Rating Scale–Revised (ALSFRS-R) (8). The ALSFRS-R is a validated, widely used instrument that evaluates functional impairment across bulbar, motor, and respiratory domains. Changes in ALSFRS-R scores from baseline to each assessment point will be analyzed, with the primary comparison defined as the slope of decline from baseline to 12 months between the intervention groups.

Secondary outcomes are designed to capture additional dimensions of therapeutic impact, including disease progression, neuromuscular performance, respiratory function, and patient-reported quality of life. Longitudinal changes in ALSFRS-R across all follow-up visits will provide an index of disease trajectory. Limb function will be evaluated using the Manual Muscle Test (MMT) to assess muscle strength and the Modified Ashworth Scale (MAS) to quantify spasticity. Respiratory function will be assessed using forced vital capacity (FVC), vital capacity (VC), forced expiratory volume in 1 second (FEV1), the FEV1/FVC ratio, peak expiratory flow (PEF), and maximal voluntary ventilation (MVV), standardized to predicted values based on age, sex, and height. Quality of life will be measured using the ALS Assessment Questionnaire-40 (ALSAQ-40), which covers physical mobility, activities of daily living, emotional well-being, and social engagement. Fiberoptic Endoscopic Evaluation of Swallowing (FEES) will be used as the primary method for evaluating swallowing function. FEES is a validated, gold-standard tool that allows real-time visualization of the pharyngeal phase of swallowing. FEES will be used for baseline assessment and regular follow-up evaluations. This will enable us to track the progression of swallowing dysfunction throughout the course of ALS and assess the impact of the intervention on bulbar function. In the nested imaging substudy, presynaptic integrity will be evaluated using ^18^F SynVesT-1 PET at baseline and Month 6, with standardized uptake value ratios (SUVR) in motor system regions serving as mechanistic biomarkers. Exploratory analyses will also examine correlations between synaptic PET changes and clinical measures (ALSFRS-R, respiratory outcomes, MMT, MAS, and ALSAQ-40).

### 3.7 Adverse events

In accordance with International Conference on Harmonization (ICH) Good Clinical Practice guidelines, a serious adverse event (SAE) is defined as any untoward medical occurrence that results in death, is life-threatening, requires inpatient hospitalization or prolongation of existing hospitalization, results in persistent or significant disability/incapacity, or leads to a congenital anomaly or birth defect. Any other medically significant event that may jeopardize the patient, or may require medical or surgical intervention to prevent one of these outcomes, will also be classified as an SAE.

Patient safety will be closely monitored throughout the study. Safety assessments will include routine physical examinations, monitoring of vital signs, laboratory tests, and 12-lead electrocardiograms (ECGs). All adverse events (AEs), regardless of severity, duration, or suspected relationship to study intervention, will be systematically documented in the electronic case report form (eCRF), reviewed by the investigator, and followed until resolution or stabilization. All SAEs will be reported promptly to the Ethics Committee in compliance with regulatory requirements and institutional policies. The Data and Safety Monitoring Board (DSMB), where applicable, will periodically review safety data to ensure ongoing participant protection.

### 3.8 Statistical analysis

All analyses will be performed using SPSS version 25.0 and R. Continuous variables will be checked for normality with the Kolmogorov–Smirnov test and summarized as mean ± SD or as median (interquartile range) if not normally distributed. Between-group comparisons will use analysis of variance (ANOVA) or Kruskal–Wallis tests as appropriate, while categorical variables will be expressed as counts and percentages and compared with chi-square or Fisher’s exact tests. The primary endpoint, ALSFRS-R slope from baseline to 12 months, will be analyzed using a mixed-effects model for repeated measures including group, time, and group × time interaction, with baseline score as covariate and unstructured covariance for within-subject correlation. Secondary outcomes such as muscle strength, spasticity, respiratory function, and quality of life will be analyzed with similar mixed-effects approaches, supplemented by post-hoc tests with Bonferroni correction when necessary. For the imaging substudy, changes in SV2A PET SUVR values will be analyzed using the same mixed-effects framework, with multiplicity controlled across primary regions of interest. Correlations between imaging and clinical outcomes will be examined using Pearson or Spearman coefficients depending on distribution. Analyses will follow the intention-to-treat principle, with missing data handled using maximum likelihood estimation under a missing-at-random assumption, and sensitivity analyses performed to test robustness. A two-sided *p* value <0.05 will be considered statistically significant for primary analyses.

### 3.9 Data management

An electronic data capture (EDC) system will be used for data entry, verification, storage, and overall trial data management. The electronic case report form (eCRF), designed in accordance with the Ethics Committee–approved template, will serve as the primary instrument for clinical data collection. All source documents, including clinical assessments, laboratory reports, imaging data, and signed informed consent forms, will be securely archived at the study site. Data entered into the eCRF must be consistent with, and verifiable against, the original source documents. To ensure data integrity and reliability, quality control procedures such as double data entry, audit trails, and automated range and logic checks will be implemented. Data handling will follow the principles of Good Clinical Practice (GCP) and comply with applicable Chinese regulatory requirements. Access to the EDC system will be restricted to authorized study personnel through role-based permissions and password protection, ensuring confidentiality and traceability of all entries. Patient safety and trial integrity will be safeguarded by continuous monitoring. A Data Monitoring Committee (DMC) will review accumulating data at predefined intervals, evaluate safety signals, and provide recommendations on study continuation or modification.

## 4 Discussion

ALS remains a devastating neurodegenerative disorder with limited therapeutic options. Although Riluzole modestly prolongs survival, its impact on functional decline is minimal, and no treatment to date has demonstrated robust disease modification (8, 9). This underscores the need for adjunctive strategies that can preserve motor function, alleviate symptoms, and improve quality of life. Tuina therapy offers such potential. Evidence suggests that Tuina may improve muscle strength, reduce spasticity, alleviate pain, and enhance circulation—symptoms that are highly relevant to ALS (16, 23, 24). Preclinical findings further support its role in modulating neuroplasticity and preserving synaptic integrity. Animal models indicate that Tuina can influence motor neurons, stabilize neuromuscular junctions, and promote vascular and glial responses (14, 25, 26). These mechanisms provide a theoretical basis for Tuina’s potential to preserve synaptic integrity—a key pathological site in ALS (27). The present trial is designed to evaluate whether integrating Tuina with Riluzole can slow functional decline and enhance quality of life in early-stage ALS. Importantly, the inclusion of a presynaptic SV2A PET substudy enables mechanistic exploration of whether clinical benefits are accompanied by measurable preservation of synaptic density in motor regions (28). This combined clinical–mechanistic approach will provide valuable insights into the neurobiological basis of Tuina’s effects, bridging complementary medicine with modern neuroimaging biomarkers. This study is expected to make a meaningful contribution by providing the first rigorous randomized evidence on Tuina therapy in ALS, both in terms of clinical outcomes and mechanistic biomarkers. If successful, it could support the integration of Tuina into comprehensive ALS management and pave the way for larger, multicenter confirmatory trials.

## 5 Ethics and dissemination

This study protocol has received ethical approval from the Ethics Committee of the Hubei Provincial Hospital of Traditional Chinese Medicine (Approval No. HBZY2022-C42-01) and is conducted at the same institution. The trial is registered with the Chinese Clinical Trial Registry (ChiCTR2300068650; registered 27 February 2023). Reporting follows the CONSORT (Consolidated Standards of Reporting Trials) and SPIRIT (Standard Protocol Items: Recommendations for Interventional Trials 2025 version) guidelines (29, 30).

## 6 Limitations

This study is conducted within certain practical constraints. Due to the nature of the intervention, complete blinding of participants and therapists is not feasible. However, robust measures have been implemented to standardize the delivery of the intervention and ensure strict adherence to the study protocol, minimizing potential performance bias. Outcome assessments will be conducted by blinded evaluators to enhance the reliability of the results. While this limitation exists, it does not detract from the significance of the study, which aims to provide valuable evidence supporting an effective and accessible therapeutic strategy for ALS management.

## 7 Conclusion

This study will provide the first rigorous evidence for the clinical efficacy of Tuina therapy combined with Riluzole in patients with early-stage ALS. By integrating a mechanistic substudy using SV2A PET imaging, it will also offer valuable insights into how Tuina may preserve synaptic integrity, potentially mitigating functional decline. If successful, the trial could pave the way for incorporating Tuina into standard ALS care as a complementary, non-pharmacological treatment, with the potential to improve quality of life and slow disease progression. The findings could stimulate larger, multicenter studies and encourage the integration of traditional Chinese medicine into Western therapeutic frameworks for neurodegenerative diseases.

## Glossary

ALS: Amyotrophic lateral sclerosis
QoL: Quality of life
PT: Physical training
FVC: Forced vital capacity
ALSFRS-R: Amyotrophic Lateral Sclerosis Functional Rating Scale–Revised
IC: Informed consent
IV: Invasive ventilation
ROM: Range of motion
MMT: Manual muscle test
MAS: Modified Ashworth Scale
VC: Vital capacity
FEV1: Forced expiratory volume in one second
FEV1/FVC: Ratio of FEV1 to FVC
PEF: Peak expiratory flow
MVV: Maximal voluntary ventilation
ALSAQ-40: 40-item Amyotrophic Lateral Sclerosis Assessment Questionnaire
SAE: Serious adverse event
AE: Adverse event
ECG: Electrocardiogram
eCRF: Electronic case report form
EDC: Electronic data capture
GCP: Good clinical practice
HIF: Hypoxia-Inducible factor
NF-κB: Nuclear Factor Kappa-Light-Chain-Enhancer of Activated B Cells
SPIRIT: Standard Protocol Items: Recommendations for Interventional Trials
CONSORT: Consolidated standards of reporting trials.
